# Filicide: Mental Illness in Those Who Kill Their Children

**DOI:** 10.1371/journal.pone.0058981

**Published:** 2013-04-04

**Authors:** Sandra M. Flynn, Jenny J. Shaw, Kathryn M. Abel

**Affiliations:** 1 Centre for Mental Health and Risk, University of Manchester, Manchester, United Kingdom; 2 Centre for Women’s Mental Health, University of Manchester, Manchester, United Kingdom; Baylor College of Medicine, United States of America

## Abstract

**Background:**

Most child victims of homicide are killed by a parent or step-parent. This large population study provides a contemporary and detailed description of filicide perpetrators. We examined the relationship between filicide and mental illness at the time of the offence, and care received from mental health services in the past.

**Method:**

All filicide and filicide-suicide cases in England and Wales (1997–2006) were drawn from a national index of homicide perpetrators. Data on people in contact with mental health services were obtained via a questionnaire from mental health teams. Additional clinical information was collected from psychiatric reports.

**Results:**

6144 people were convicted of homicide, 297 were filicides, and 45 cases were filicide-suicides. 195 (66%) perpetrators were fathers. Mothers were more likely than fathers to have a history of mental disorder (66% v 27%) and symptoms at the time of the offence (53% v 23%), most often affective disorder. 17% of mothers had schizophrenia or other delusional disorders. Overall 8% had schizophrenia. 37% were mentally ill at the time of the offence. 20% had previously been in contact with mental health services, 12% within a year of the offence.

**Conclusion:**

In the majority of cases, mental illness was not a feature of filicide. However, young mothers and parents with severe mental illness, especially affective and personality disorder who are providing care for children, require careful monitoring by mental health and other support services. Identifying risk factors for filicide requires further research.

## Introduction

Homicide is a leading cause of childhood death in the developed world. UNICEF [1] reported 3500 children die from maltreatment every year. Child deaths from maltreatment in the UK were reported as 0.9 per 100,000, approximately 2 children a week. A recent review commented, filicide “…is a problem that transcends national boundaries. Little evidence exists about factors associated with its commission and this lack of knowledge makes prevention difficult” [2]. The existing literature is challenged by a lack of methodologically robust research. The most recent national descriptive study from Japan had a large sample size. However, the sample was derived from newspaper reports of filicide, which lacked sufficient case information particularly mental health history [3].

Few studies have been able adequately to assess the relationship between filicide and mental illness in a national sample. Studies suggesting an association between filicide and mental illness are often based on small samples [4] span decades to achieve sufficient numbers [5] or are biased due to samples derived from psychiatric or forensic facilities [6]. These studies will inevitably show high incidence of mental illness whereas studies of other populations, such as prisons or child protection cases, often show lower incidence of mental illness and a higher proportion of child abuse and social disadvantage [6]. These distinctions are important because the antecedents of violent behaviour and abuse are not necessarily the same as those of mental disorder. Therefore, preventive strategies are likely to be different and to have different implications for services.

Our previous findings on infanticide suggested that the prevalence of mental illness is strongly associated with the gender of the perpetrator, with women significantly more likely to have been mentally ill at the time of offence [7]. The distinction between maternal and paternal filicide may be important in understanding risk but most studies have been unable to examine effects by gender.

To address these problems with the current literature, we used data from the National Confidential Inquiry into Suicide and Homicide by People with Mental Illness (NCI), a longitudinal national case series of all homicides in the UK, with particular focus on perpetrators with mental illness. Using this contemporary, nationally representative sample, we aimed first to, provide a detailed, up-to-date description of the demographic and forensic characteristics of filicide perpetrators; second, to measure the prevalence of mental illness among filicide perpetrators; and third to investigate the prevalence of mental illness by gender of the perpetrator.

## Methods

In this study the NCI sample used is a 10-year consecutive case series of convicted homicides and homicide-suicides offences (01/01/97-31/12/06) in England and Wales. Homicides are defined as murder, manslaughter or infanticide under the Homicide Act (1957). Homicide-suicides are defined as cases where the suicide occurred no more than 3 days after the homicide [7]. Filicides are homicides committed by a parent or adult in-loco parentis, with the victim aged under 18. Data collection occurred in three stages:

1. A total sample of homicide perpetrators was obtained from the Home Office. Filicide-suicide cases were linked using suicide data from the Office for National Statistics.
2. For all convicted homicide perpetrators, psychiatric reports were requested from courts, psychiatrists and forensic units. Psychiatric assessments conducted post offence describe: (i) symptoms of mental illness at any point preceding the offence; (ii) at the time of the offence; and (iii) onset of illness after the offence. Psychiatric reports were obtained in 195 cases (66%). Of those without reports (n = 102), the courts confirmed a psychiatric assessment was not requested in 77 cases (75%) therefore it was unlikely that these individuals had serious mental illness; in 8 (8%) the entire court file was missing, in 16 cases (16%) no reason was provided for the absence of a report. Where data from psychiatric reports is presented, the absence of a report has been coded as missing data, as although unlikely, we cannot assume those without reports did not have mental illness.
3. To identify those with a history of mental health service contact, details were submitted to hospital and community Trusts in the perpetrator’s district and adjacent districts, and a member of the mental health team was sent a questionnaire to complete relating to the care and treatment of the patient. ICD-10 diagnostic criteria were used [8] and the accuracy of the questionnaire data was high [9]. Records of previous offences were obtained from the Police National Computer.

### Ethnicity

Data defining ethnic group was provided by the Homicide Index. Ethnic group was categorised using police observation or self-reports by perpetrators and coded onto the Homicide Index. The predefined groups were: White, Black, Asian and Other.

### Statistical Analysis

Findings are reported as proportions with 95% confidence intervals. Stata 10 was used to calculate the Pearson Chi-square statistic; where numbers in cells were less than 5, Fisher’s exact test was used, and a 2-sided *P* value of <0.05 was considered significant [10]. The test value and degrees of freedom are also presented. If an item was unknown for a case, the case was removed from analysis of that item; the denominator in estimates is the number of valid cases indicating the number of missing cases per item.

### Ethical Approval

The NCI obtained ethical approval on 1 October 1996, and exemption under section 251 of the National Health Service Act (2006), permitting confidential and identifiable information to be obtained without informed consent. The study was registered under the Data Protection Act (1998).

## Results

### Demographic and Forensic Characteristics

**Figure 1:**
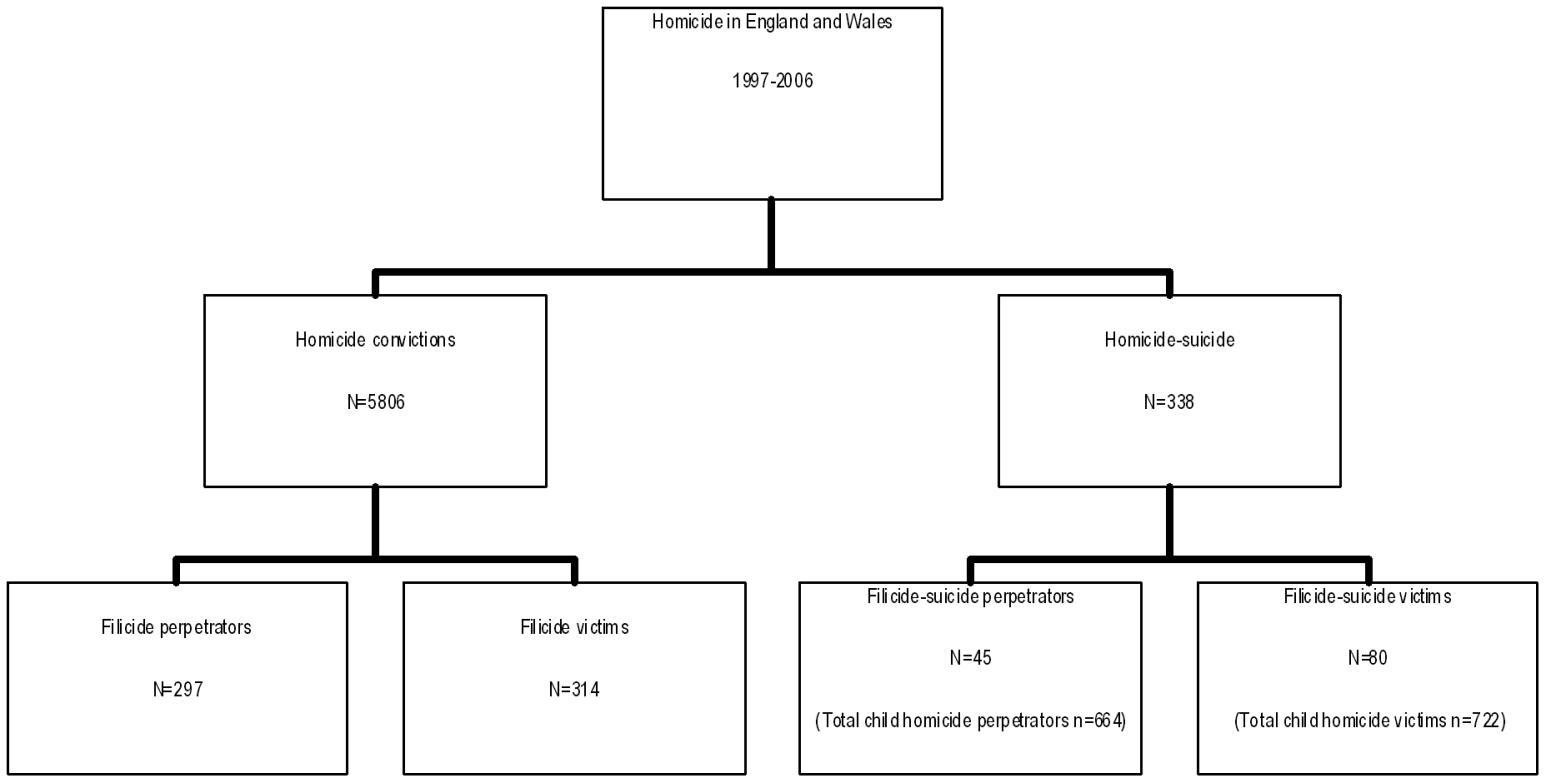
Flow chart of study sample.

In England and Wales, we identified 342 filicide perpetrators between 1^st^ January 1997 and 31^st^ December 2006, 297 (87%) were convicted of killing their child/stepchild and an additional 45 (13%) died by suicide within 3 days of the homicide (Figure 1). Filicide-suicides are referred to separately from the main analysis as clinical data from psychiatric assessments (post-offence) were unavailable. The main analyses are therefore based on 297 convicted parents/step-parents.

Overall, a significantly higher proportion of fathers than mothers were convicted of filicide; a male to female ratio = 2∶1. The median age of perpetrators was 27 years (Table 1); 47 (16%) perpetrators were teenagers at the child’s birth, of whom 22 were mothers. Sixty-three (22%) were from ethnic minorities. The most frequent method of filicide was categorised as unspecific, non-accidental injury, including shaking, skull fractures, and multiple injuries (129, 43%). This method was more common with fathers than mothers. Suffocation/smothering was the second most common method, and more frequent with mothers. Seventy-three (25%) had previous convictions for violence, significantly more fathers than mothers. Of perpetrators with a psychiatric report, 90 of 185 cases with data (49%) had a history of substance misuse; more common in fathers. There were low rates of substance dependence.

**Table 1:** Demographic and forensic characteristics of perpetrators convicted of filicide and their victims.

	Paternal FilicideN = 195 valid % CI			Maternal FilicideN = 102 valid % CI			Total FilicideN = 297 valid % CI			p-value	X^2^ value	Degrees of freedom
**Demographic characteristics:**												
Age of Perpetrator: Median (range)	27	(15–70)		27	(14–50)		27	(14–70)		0.19		
Unmarried (not cohabiting)	19	18	11–26	49	56	45–67	68	35	28–42	<0.01	31.8231	1
Unemployed/long term sick	61	62	51–71	46	60	48–71	107	61	53–68	0.80	0.2672	1
Ethnic minority group	34	18	13–24	29	29	20–39	63	22	17–27	0.04	4.4509	1
**Behaviour characteristics:**												
Convictions for violence	60	32	25–39	13	13	7–21	73	25	20–31	<0.01	12.9006	1
History of alcohol misuse	28	29	21–40	19	24	15–35	47	27	21–34	0.42	0.6434	1
History of drug misuse	47	47	37–58	24	30	20–41	71	40	32–47	0.02	5.6457	1
**Method of filicide**												
Unspecific non-accidental injury	103	53	46–60	26	25	17–35	129	43	38–49	<0.01	20.3602	1
Suffocation/smothering	15	8	5–13	19	20	12–29	34	12	8–16	<0.01	8.4148	1
Hitting and/or kicking	19	10	6–15	2	2	0–6	21	7	5–11	0.02	5.9738	1
Sharp instrument	13	7	4–12	4	4	1–10	17	6	4–9	0.44	0.8529	1
Strangulation	9	5	2–9	6	6	2–13	15	5	3–9	0.60	0.2718	1
Blunt instrument	7	4	2–8	3	3	1–9	10	4	2–4	1.00	0.0670	1
**Outcome:**												
Murder	84	43	36–50	9	9	4–16	93	31	26–37	<0.01	36.5330	1
Manslaughter	101	52	45–59	53	52	42–62	154	52	46–58	0.98	0.0007	1
Manslaughter (Sec 2)	8	4	2–8	17	17	10–25	25	8	6–12	<0.01	13.7134	1
Infanticide 1	0	0	0–2	21	21	13–30	21	7	4–11	<0.01	43.2017	1
Not guilty by reason of insanity/unfit to plead	2	1	0–4	2	2	0–7	4	1	0–3	0.43	0.4408	1
**Disposal:**												
Imprisonment	180	92	88–96	35	34	25–44	215	72	67–77	<0.01	112.6950	1
Hospital disposal	9	5	2–9	26	25	17–35	35	12	8–16	<0.01	28.0716	1
Community based sentence	6	3	1–7	41	40	31–50	47	16	12–20	<0.01	69.2707	1

1 Infanticide verdicts only apply to women.

### Victim Characteristics

**Figure 2:**
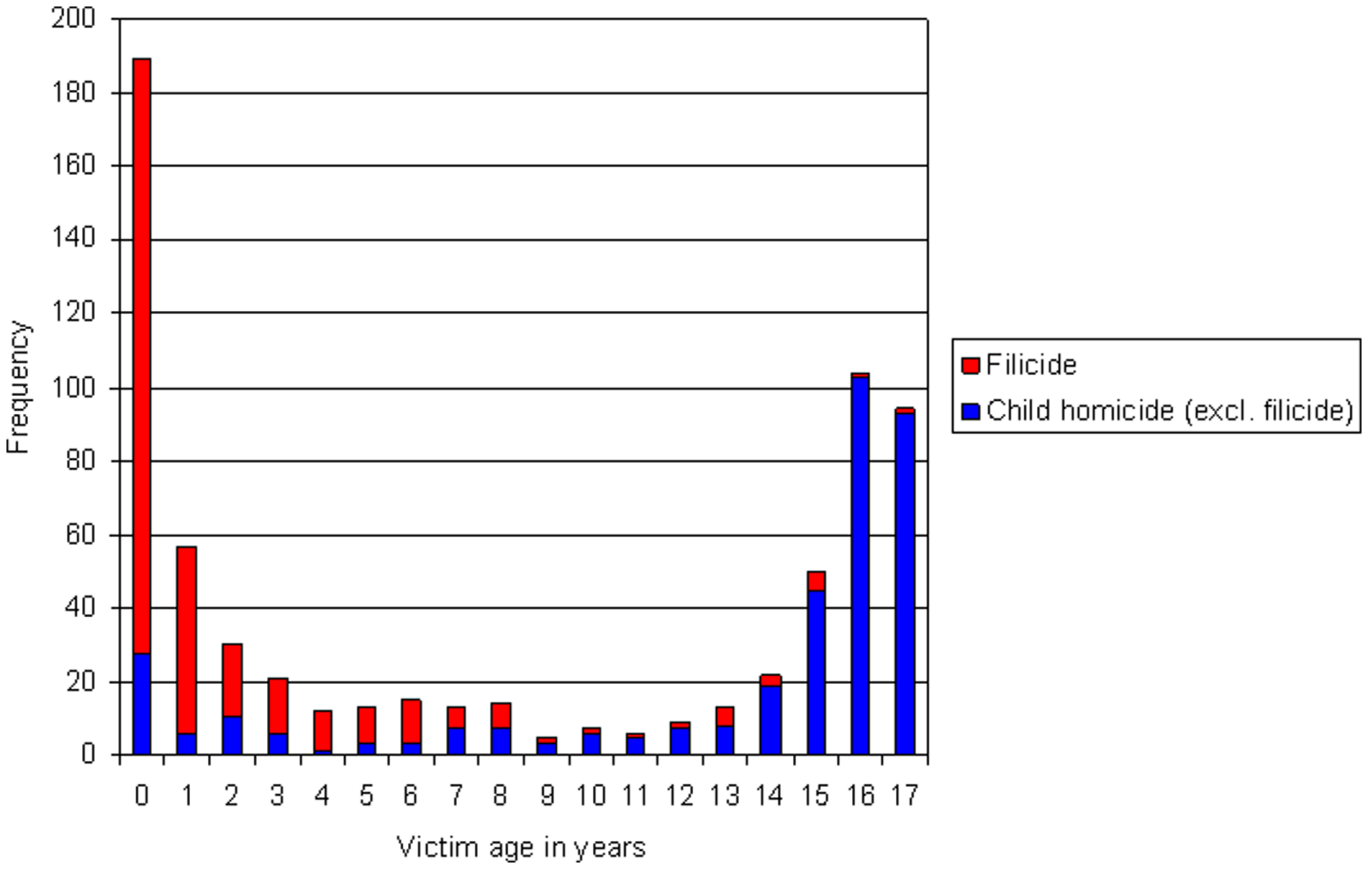
Frequency of filicide and other child homicide cases, by age of victim.

There were 314 victims; 162 (52%) boys and 152 (48%) girls. Over half were infants (161, 51%). Seventy-three (23%) were killed in the first 3 months of life, including 13 neonaticides (<24 hours old). Most infants were killed by fathers (104, 65%). One hundred and seven victims (34%) were preschool age (1–5 years), and 46 (15%) were between 6 and 17 years. Figure 2 shows the distribution of victim age for all filicide victims (n = 314) and a comparison with other child homicide victims (n = 408) during the same time period. Young children were more commonly killed by parents, whereas teenagers were more often killed by others known to them.

### History of Mental Illness

One hundred and nineteen perpetrators (40%) had a history of mental health problems; more common in maternal than paternal perpetrators (67, 66% v 52, 27%) Table 2. Overall, the most common diagnosis was affective disorder (43, 14%). Severe mental illness (affective disorder or schizophrenia) occurred in 68 cases (23%). Of those with psychiatric reports (195, 66%), 64 of 173 with data (37%) were mentally ill at the time of the offence, most often experiencing depression. Mothers were more likely to have symptoms of mental illness at the time of the offence (42, 53% v 22, 23%), particularly depressive symptoms. Of all perpetrators experiencing symptoms prior to filicide, 19 (of 168 with data, 11%) experienced delusions, in 13 (8%) these related to the victim and 9 (5%) experienced delusions with violent content at the time of the homicide. Seventeen (of 170 with data, 10%) experienced hallucinations, of which 4 were specifically about the victim (Table 2). Twenty (10%) had co-morbidity (severe mental illness and substance dependence and/or misuse.

**Table 2:** Clinical characteristics of perpetrators convicted of filicide.

	Paternal FilicideN = 195 valid % CI			Maternal FilicideN = 102 valid % CI			Total FilicideN = 297 valid % CI			p-value	X^2^ value	Degrees of freedom
**Clinical characteristics:**												
Any contact with psychiatric services	26	13	9–19	32	31	23–41	58	20	15–25	<0.01	13.8676	1
Contact in the last year	14	7	4–12	21	21	13–30	35	12	8–16	<0.01	11.5824	1
Lifetime history of mental illness	52	27	21–33	67	66	56–75	119	40	34–46	<0.01	42.4610	1
Schizophrenia & other delusional disorders	8	4	2–8	17	17	10–25	25	8	6–12	<0.01	13.7134	1
Affective disorder	15	8	4–12	28	27	19–37	43	14	11–19	<0.01	21.1157	1
Personality disorder	16	8	5–13	14	14	8–22	30	10	7–14	0.13	2.2475	1
Alcohol dependence	5	3	1–6	1	1	0–5	6	3	1–7	0.67	0.8486	1
Drug dependence	5	3	1–6	3	3	1–8	8	3	1–5	1.00	0.0363	1
Co-morbidity (severe mental illness and alcohol and/or drug dependence/misuse)	10	9	5–17	10	11	6–20	20	10	6–15	0.68	0.1707	1
**Symptoms of mental illness: data from psychiatric reports:**	**N = 102 valid % CI**			**N = 83 valid % CI**			**N = 185 valid % CI**			**p-value**	**X^2^ value**	**Degrees of freedom**
Mentally ill at the time of the offence	22	23	15–33	42	53	42–64	64	37	30–44	<0.01	16.7018	1
Depression at offence	12	13	7–22	34	45	33–57	46	27	21–34	<0.01	21.7631	1
Psychosis at offence	11	12	6–20	14	18	10–29	25	15	10–21	0.21	1.5835	1
Hallucinations at offence	7	7	3–15	10	13	7–23	17	10	6–15	0.21	1.5807	1
Delusions at offence	7	7	3–15	12	16	9–27	19	11	7–17	0.07	3.2634	1
**Data from questionnaires:**	**N = 26 valid % CI**			**N = 32 valid % CI**			**N = 58 valid % CI**			**p-value**	**X^2^ value**	**Degrees of freedom**
In-patients	2	8	1–27	0	0	0–0	2	4	0–13	0.19	2.6808	1
Post-discharge patients	1	5	0–24	3	10	2–26	4	8	2–19	0.64	0.4260	1
Symptoms at last contact with services	14	64	41–83	16	53	34–72	30	58	43–71	0.46	0.5520	1

### Receiving Mental Health Services

Fifty-eight (20%) had been under the care of mental health services, 35 (12%) within 12 months of the offence (Table 2). Of those 35 patients in recent contact with services, 19 (of the 33 who responded; 58%) had previous in-patient admissions (9 fathers, 10 mothers). Two were in-patients at the time of the offence. Four perpetrators committed filicide within 3 months of discharge from in-patient care. Of those with symptoms of mental illness at the time of the offence (64 cases), 5 had previously been treated, but not in the last year, 40 (63%) had no previous contact with services.

### Biological and Step-parents

Two hundred and thirty-seven perpetrators (80%) killed a ‘*biological*’ child/children. Of the 60 perpetrators who killed a step-child, 1 (2%) was killed by a step-mother; 59 (98%) by a step-father; no adoptive parents committed filicide. An analysis was undertaken to examine whether there were any demographic, behavioural, offence or clinical differences between fathers and step-fathers who committed filicide. There were no demographic differences, however step-fathers were more likely to have a history of substance misuse (27, 39% v. 20, 67%; p = 0.01); to kill victims of preschool age (26, 19% v. 34, 58%; p<0.01) and less likely to kill infants (88, 65% v. 13, 22%; <0.01). Step fathers more commonly used hitting and kicking as a method of homicide (7, 5% v 12, 21%; p<0.01), and were less likely to use ‘other methods’ associated with infant deaths, such as shaking (80, 59% v. 23, 39%; p = 0.01). Step-fathers were also more likely to be convicted of murder (48, 35% v 36, 61%; p<0.01), less likely to be convicted of manslaughter (80, 59% v. 21, 36%; p<0.1) and more commonly received a prison disposal (122, 90% v. 58, 98%; 0.04). Biological fathers were more likely to have been mentally ill at the time of offence (20, 30% v. 2, 7%; p = 0.02), and to have depressive symptoms at the time of offence (12, 18% v. 0, 0%; p = 0.02).

### Filicide-suicide

**Figure 3:**
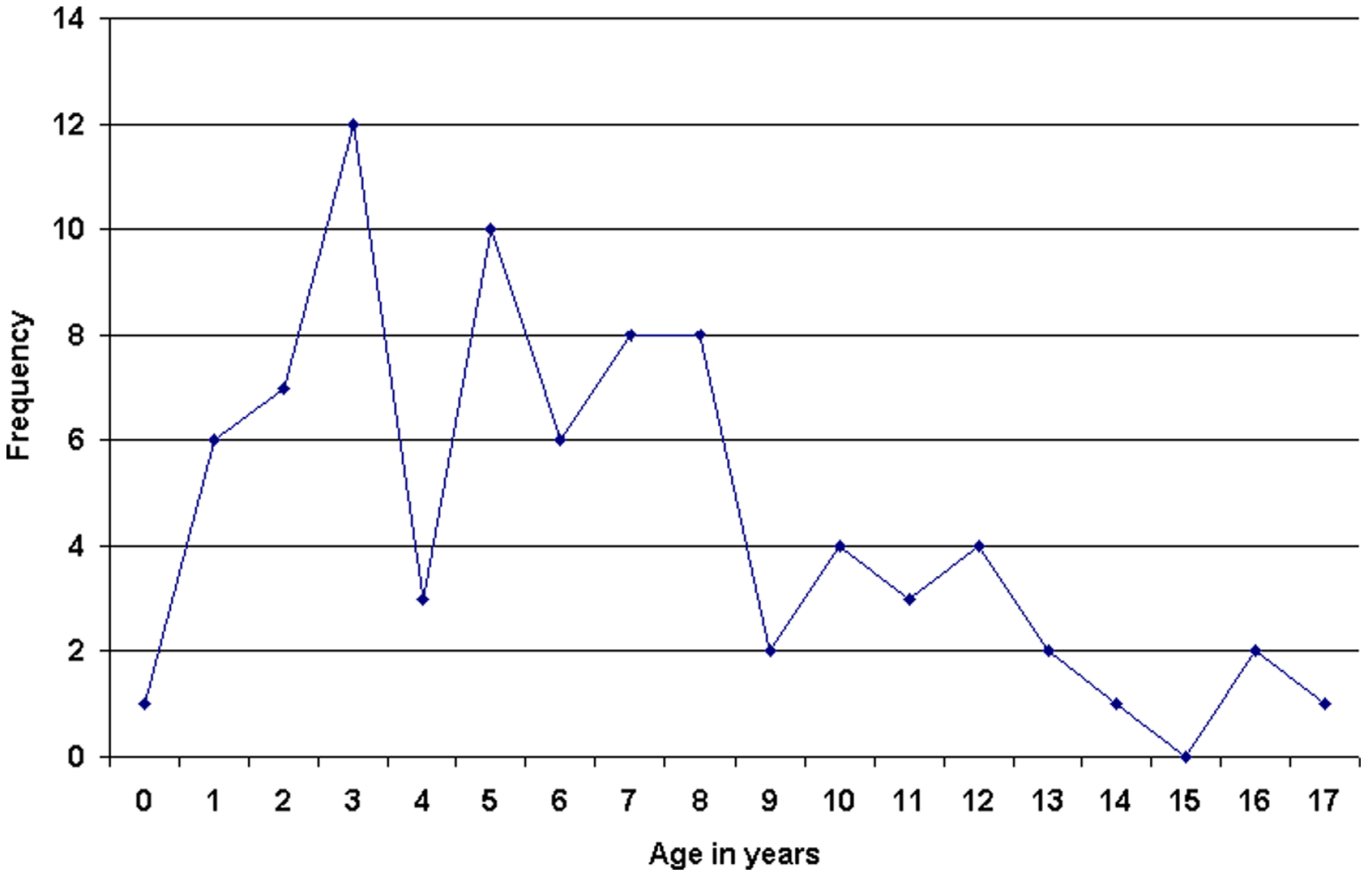
Frequency of filicide-suicide cases, by age of victim.

Of 45 filicide-suicide perpetrators, 28 (62%) were men. The median age was 37. Multiple victims were common in filicide-suicide, with 80 child victims in total. In 14 cases, a spouse/partner or ex-spouse/partner was also killed. The median age of the 80 filicide-suicide victims was 6 years (Figure 3). Methods of homicide included; exhaust fumes (8 incidents, 17 deaths), other poisoning (6 incidents, 7 deaths), sharp instrument (5 incidents, 8 deaths), strangulation/suffocation (8 incidents, 10 deaths) and arson (4 incidents, 15 deaths). Five perpetrators (10%) had previous contact with mental health services, 2 (4%) within 12 months of the offence.

## Discussion

### Mental Illness

The overrepresentation of mental illness in filicide is the key finding of this study; 40% of perpetrators had a recorded mental disorder, which is consistent with findings from smaller studies [11], [12]. The most common diagnoses were affective disorders and personality disorder; not psychosis, which may contrast with popular perception, and that of some professionals caring for mentally ill parents [13]. The proportion of perpetrators with psychosis was still high at 15%, (18% mothers) compared to 6% of homicides in general population studies (NCI 2011) [14]. However, previous studies have emphasised higher prevalence of psychotic disorder in filicide perpetrators, which may largely be due to sample bias. Although, in our perpetrators, the proportion with psychosis was markedly lower than in some studies, the rate remains significantly higher than the general population: 0.4% overall prevalence in UK, 0.5% for women and 0.3% for men, with highest rates in people aged 35–44 years for women and men (1.1% and 0.7% respectively) [15].

### Receipt of Psychiatric Care

In this study, less than half the perpetrators with mental illness had previous contact with mental health services, fewer fathers than mothers. Of the total, 20% were treated before the offence, which is lower than a similar Swedish population study (35%) [16]. Overall, our findings are consistent with those reported from a similar large, population-based sample in Denmark, where, most parents committing filicide had no prior psychiatric history. However, Laursen et al (2011) did report a 9-fold excess risk of child homicide among children whose parents were previously admitted to psychiatric hospital, compared to those with no admission, although they were limited to register records of in-patient admission so that only more severe cases of illness in the parents were examined [17].

### Demographics

From this large population-based nationally representative case series, we report that the median age of female perpetrators was 27 years. The majority of women killed an infant or child aged 1 or younger. Therefore, in most cases, their age was consistent with the average age of a woman at first live birth in England and Wales [18] and consistent with Overpeck et al’s 1998 study of infanticide [19]. Twenty-three percent of maternal perpetrators were teenagers at the victim’s birth. In the general population, the proportion of babies born to a teenage mother was 7% (average percentage for 1999–2006) [18]. A wealth of literature has examined the association between becoming a parent at a young age and future life outcomes. Our recent study reported a significantly greater risk of premature death from all causes [20], but there is inconsistent evidence of an increase in severe physical aggression by younger parents [21]. The risk in young parents appears to be elevated where multiple young children live in the household [22]. Overall, it is likely to be the case that young mothers live under more difficult and deprived circumstances compared to those who delay childbearing, they have poorer educational attainment, economic opportunities and marriage stability. Therefore these parents are at a greater risk of poor outcomes themselves than of inflicting poor outcomes on their infants.

Eighty percent of filicide perpetrators were biological parents. There were differences between fathers and step-fathers, with step-fathers more likely to kill preschool children than infant victims. This is consistent with previous research in a British sample of filicide perpetrators which has shown that child abuse is predominantly perpetrated against children under the age of 5. Cavanagh et al., (2007) in a study of 26 cases revealed that 60% of birthfathers killed infants, whereas only 6% of stepfathers killed an infant. In 94% of cases where children were killed between the ages of 1–4, the perpetrators were stepfathers [23]. Stepfathers were also less likely to have mental illness, and more commonly having a history of drug misuse. A fifth of all perpetrators were from a minority ethnic group, a higher proportion than represented in the general population (8%). This finding suggests an overrepresentation of ethnic minority groups in filicide, consistent with a previous study of child homicide [24].

Fathers were significantly more likely to be perpetrators than mothers; they were also more likely to use violent methods of killing; have previous convictions for violent offences; perpetrate multiple killings, and have a history of substance misuse/dependence. Victims were equally likely to be girls as boys. Infants were most likely to be victims rather than school aged or older children. The relative risk of homicide in children by parents with a history of psychiatric admissions was shown to be markedly higher in children age 1–4 years (9.8 CI 4.91–19.57) in a recent Danish register study [25]. The relative risk of homicide reduced markedly in those age 5–15 years, 5.15 (CI 2.51–10.55).

### Filicide-suicide

We had access to a unique database, compiled by the NCI, which enabled analysis on rare events such as filicide-suicide in a national sample. Of the total filicide cases recorded, we found 13% of perpetrators took their own life after killing their child. Consistent with previous research by Friedman et al (2005), we found men committed almost twice the number of filicide-suicides [26]. ‘Familicide’, which include spouses and child victims, was recorded in a third of the filicide-suicide cases. Few perpetrators were under the care of mental health services within a year of the offence; a much smaller proportion than previously reported in a US sample [26]. This finding would suggest that the perpetrators were either not experiencing serious mental health problems at the time or, that they had not sought help.

### Strengths and Limitations

The scope of the NCI’s clinical dataset enables analysis of serious adverse outcomes, such as filicide, in greater detail than other epidemiological studies. There is no other comparable system of data collection, that we are aware of, which includes such a wide group of filicide perpetrators; those in contact with mental health services; people with mental illness at the time of the offence; and those with no history of mental disorder. The 10-year period of monitoring provided a contemporary dataset, which enabled the examination of factors likely to be currently associated with filicide.

However, there were methodological limitations. We are unable to draw aetiological conclusions because we do not have a comparison group. To place the findings in context, we used population denominators where available on mental illness (psychosis and depression), childbearing age, and ethnicity in the general population as a comparator. Information was obtained from case records and clinical judgement as opposed to standardised assessments, and clinicians were not blind to outcome, which may have generated bias. Our data only included convicted homicide perpetrators, and those suspected of homicide who committed suicide. This may underestimate actual cases of intentional child deaths, as it does not include convictions for child cruelty (where ‘*homicide’* liability was not proven). Similarly, a history of violence is derived from incidents resulting in conviction; therefore, the true extent of ‘all’ previous violent behaviour may be underestimated. Information on other relevant variables was not routinely available in all psychiatric reports, e.g. social deprivation, learning difficulties, domestic violence, and child abuse.

Identifying associations between mental illness and filicide has clear implications for service providers, requiring greater vigilance of patients who are parents. This is increasingly relevant because better care means more people with mental illness are becoming parents [27]. Violence and homicide involving children has been linked to unemployment, deprivation and poverty, as well as lone parenting, insufficient social support and poor coping strategies [5], [19]; all affect mentally ill parents more [13]. Social trends in the UK show a sizeable proportion of the general population under economic stress, single parents and on low incomes [18]. This suggests that generating effective child violence/homicide prevention strategies requires broad public health approaches. Targeting sub-populations by providing high quality evidence on factors such as mental disorder and contact with mental health services may prove more constructive for health service development and violence prevention and may offer possibilities to recognise and intervene with specific risk factors [2].

These findings have implications for clinicians, particularly those caring for mothers with affective disorder. In England, 7.5% of people aged 16–64 in the general population had severe depressive symptoms requiring treatment [15]. In our sample, consistent with previous reports [2], [11], [12] affective disorder was the prevailing diagnosis in mothers (27%) and fathers (8%), and more common than psychosis, although psychotic depression may represent a particularly high risk group.

Risk-assessment of mothers in joint psychiatric care reported greatest risk of *actual* harm to child was presented by mothers with affective diagnoses rather than schizophrenia [13]. Despite this, staff consistently perceived and rated schizophrenia mothers to be the greatest risk to their infants and this was reflected in significantly higher rates of social service supervision on discharge compared to other ill mothers.

### Implications of the Study

Our findings indicate that fathers with a history of substance misuse, violence or affective disorder and mothers who were teenagers at the birth of their child, or with affective disorder may be appropriate targets for intervention. Parents with mental illness should be asked about violent thoughts toward their children, particularly if depressed. In women with postpartum depression, Jennings et al [28], reported 41% admitted to such feelings compared to 7% of controls. However, psychiatrists underestimated the extent to which depressed mothers thought about harming their child in a recent US study [29]. Such thoughts are common in depressed mothers and an affirmative answer does not necessarily imply increased risk. Jennings et al [28], suggested where thoughts about harming their child were present, clinicians should explore the intensity and frequency of violent thoughts, in conjunction with psychotic symptoms, and problems with anger control.

Our results corroborate previous small studies reporting that filicide was more common in the first year of life [17]; over half of cases in this study were infants. The high proportion of infant deaths strengthens calls for early assessment detection of post-partum mental illness, which is becoming more widespread. The Pregnancy Risk Assessment Monitoring System routinely surveys women before, during and after pregnancy across 37 US states, enabling health workers to identify problems and offer services prenatally. However, few mothers reporting a perceived need for intervention with domestic violence during pregnancy received help [30].

Understanding the risk factors for filicide and the widespread nature of child abuse is far from complete. Future research on filicide should study these acts in the context of child abuse and domestic violence to support the development of effective interventions. Case control and psychological autopsies are useful future methodologies for examining risk factors and the role of mental illness in this tragic outcome. We concur with others [17] in recommending the need for more high quality, large scale population studies allowing detailed subgroup analysis and the identification of causal mechanisms.
